# Circular valorization of green leafy side streams via pressurized liquid extraction techniques for recovery of bioactive compounds

**DOI:** 10.1016/j.fochx.2025.102944

**Published:** 2025-08-23

**Authors:** Pamela F.M. Pereira, Amparo Jiménez-Quero

**Affiliations:** Division of Industrial Biotechnology, Department of Life Sciences, Chalmers University of Technology, 412 96 Gothenburg, Sweden

**Keywords:** Antimicrobial, Pectins, Bioactive extracts, Green leaves, Pressurized liquid extraction

## Abstract

Vegetable side streams are resulting non-edible by-products from vegetable processing. These side streams are a rich source of bioactive compounds and macromolecules. Despite their potential for high-value applications, these materials are frequently used in low-value applications or discarded, contributing to resource depletion and environmental concerns. This study investigates the valorization of vegetable side streams, specifically artichoke, broccoli, cauliflower, and spinach leaves, through pressurized liquid extraction (PLE) techniques aimed at recovering bioactive extracts. Two PLE methods were compared: low-pressure hot water extraction (LPHW) and sequential subcritical water extraction (s-SWE). The s-SWE method yielded higher cumulative extraction and demonstrated superior efficiency in recovering carbohydrates content, particularly pectins. It also produced extracts with enhanced bioactivity, notably great antioxidant activity due to increased phenolic compound content. Antimicrobial activity varied with the extraction method and it as biomass-dependent: LPHW extracts showed efficacy against Gram-positive for cauliflower leaf extracts, while s-SWE extracts were moderately active against both Gram-positive and negative bacteria strains for artichoke leaf extracts. These findings highlight the feasibility of pressurized liquid extraction as a non-conventional and sustainable technique for producing functional extracts from agro-food waste. Moreover, extract profiles can be tailored according to specific biorefinery goals, reinforcing the potential of vegetable side streams in circular economy strategies.

## Highlights

• Two PLE methods for valorization of vegetables by-products were compared.
• Pectin extraction was more pronounced in s-SWE.
• The phenolic profiles of the extracts varied depending of the extraction conditions and method.
• LPHW-extracted cauliflower leaves showed the highest antimicrobial activity observed.
• Artichoke leaf extracts demonstrated a balance between antioxidant and antimicrobial activities.

## Graphical abstract

**Figure:**
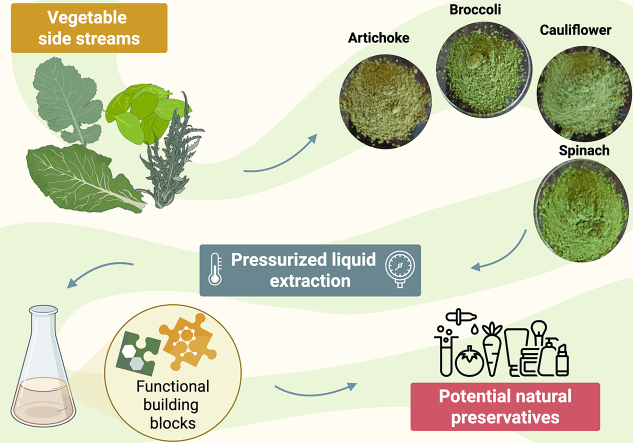

## 1 Introduction

Fruits and vegetables have great importance to the human diet as nutrient-dense foods. They provide a wide range of nutrients, phytochemicals, and fibers required for the good homeostasis and reduction of chronic diseases risk (Harris et al., 2023). As highly perishable food products, fruits and vegetables contribute significantly to high food loss generation until the distribution stage of the food value chain, second only to the group of roots, tubers, and oil-bearing crops (FAO, 2019). In Europe alone, fruits and vegetables contributed largely to food wastage, representing a coefficient of approximately 19 % on primary production and around 22.1 % at the consumption stage (De Boni et al., 2022). According to Caldeira et al. (2019), considering the inedible part, vegetables wastage represents 46 % of total available vegetables, while fruits reach the amount of 41 % of total produced.

The massive production of fruit and vegetables waste has negative impacts at societal, environmental, and economic levels, and therefore is a global issue. For this reason, food wastage mitigation strategies, including value-adding approaches, have been extensively evaluated as an efficient resource usage in the framework of the circular economy (Donner & De Vries, 2023; Ganesh et al., 2022). Vegetable loss and waste represents a great opportunity for recovery not only of nutraceuticals, phytochemicals (such as phenolic compounds), pigments (such as anthocyanins, carotenoids, and betalains), and flavoring agents (such as essential oils), but also macromolecules as carbohydrates, proteins, and lipids, which can be applied to pharmaceuticals, cosmetics, food, and materials industries.

Extractive methods for vegetables matrices valorization include Soxhlet and maceration, which are performed conventionally using organic solvents. The main drawbacks related to conventional extraction methods are related to the long extraction time required, the amount of organic solvent waste, low extraction efficiency, among others (Zhang et al., 2020). Among emerging extraction methods, pressurized liquid extraction (PLE) is an environmentally friendly method. The main advantages of PLE in comparison with conventional methods include faster processing, improved extraction efficiency and selectivity, the use of green solvents (e.g., water and ethanol), and reduced solvent consumption (Višnjevec et al., 2024). In PLE, the use of water as solvent represents the greenest solvent, offering clean processing and pollution prevention. Under pressurized conditions, water properties can be tailored above its boiling point (T_b_ 100 °C) and below the critical point (T_c_ 374 °C) combined with pressures enough to maintain its liquid state (between 0.1 and 22.1 MPa) (Plaza & Turner, 2015; Zhang et al., 2020). At subcritical state, water has increased capacity to solubilize compounds through an improved diffusivity effect, besides an increased disruption of cohesive (solute-solute) and adhesive (solute-matrix) interactions. Another interesting point is that under increasing temperature, the polarity of pressurized water tends to decrease, mimicking dissolution behavior of organic solvents (Yabalak et al., 2023).

Previous studies have demonstrated the efficacy of applying PLE for recovering bioactive compounds from vegetable matrices (Krstić et al., 2023; Pagano et al., 2018; Smiderle et al., 2017). Sequential subcritical water extraction (s-SWE) is a valuable tool for selective biomass fractionation since the extraction process can be molded according to the target compounds solubility or hydrolytic affinity ratio in a series of time or temperature (Lachos-Perez et al., 2020). Rincón et al. (2021) demonstrated the increasing temperature effect for short time on s-SWE for recovery of pectin from bay pruning waste. Lachos-Perez et al. (2020) fractionated orange peels by increasing temperature in s-SWE to obtain a bioactive-rich fraction constituted by hesperidin and narirutin, followed by the biomass hydrolytic reaction for extraction of sugars. In another study, Martinez-Fernandez et al. (2023) reported the temperature increase in a two-stage s-SWE favored the extraction of bound phenolic compounds with intact functionality. These reports demonstrated the versatility of s-SWE in the biorefinery context to maximize the potential use of agro-industry biomasses to recover value-added products.

Although comparisons with traditional extraction methods have been reported in studies involving PLE (Derakhshan et al., 2025; Park et al., 2022), a comparative investigation between PLE performance under single-step extraction and low-pressure conditions and s-SWE remains largely unexplored. This study addresses this gap by evaluating the feasibility of pressurized liquid extraction using only water as a solvent under low-pressure and subcritical conditions to recover bioactive compounds from vegetable by-products of the agro-industry, including artichoke, broccoli, cauliflower, and spinach leaves. The impact of the extraction process on yield was assessed, and a screening approach was applied to identify value-added compounds across the different vegetable extracts.

## 2 Materials & methods

### 2.1 Materials

Artichoke (*Cynara scolymus*), broccoli (*Brassica oleracea* var. *italica*), cauliflower (*Brassica oleracea* var. *botrytis*), and spinach (*Spinacia oleracea*) leaves were kindly provided by Proexport (Murcia, Spain). All biomasses were stored at −20 °C until the usage moment. Chopped biomasses were submitted to freeze-drying, followed by griding process and sieving (particle size lower than 500 μm), prior characterization.

## 3 Methods

### 3.1 Sequential subcritical water extraction (s-SWE)

**Fig. 1:**
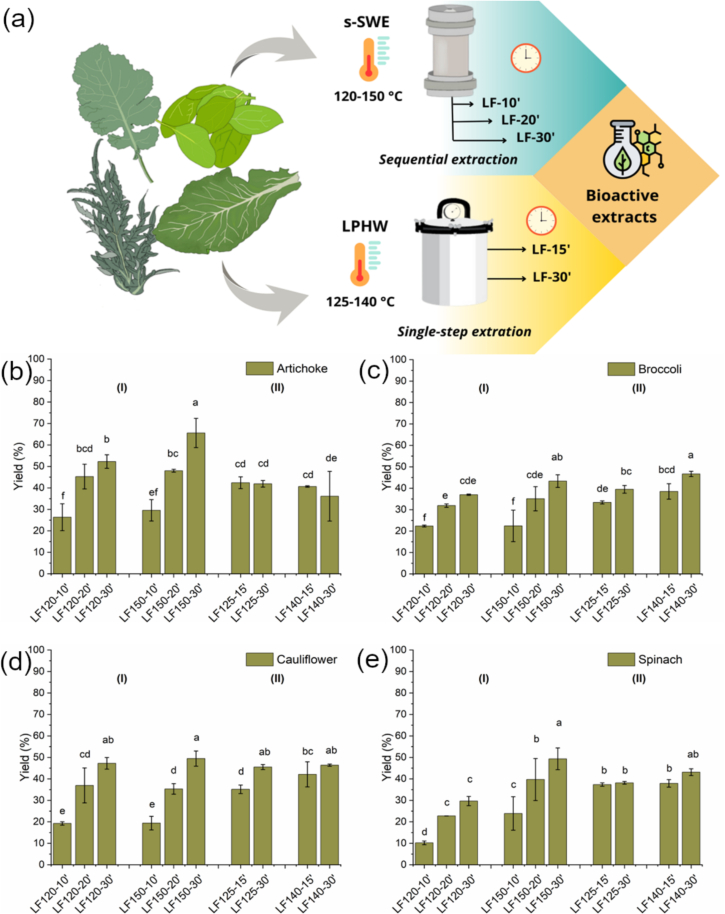
Schematic representation of the extraction processes (s-SWE and LPHW) applied to different green leaves for recovery of bioactive extracts (a) and extraction yield for the different green leaves extracts: artichoke (b), broccoli (c), cauliflower (c), and spinach (e) obtained by (I) s-SWE and (II) LPHW. (For interpretation of the references to colour in this figure legend, the reader is referred to the web version of this article.)

Sequential subcritical water extraction (s-SWE) was applied to biomass powders using an accelerated solvent extractor Dionex ASE 350 (Thermo Fischer Scientific Inc., USA). The extraction cell was prepared with a layer of diatomaceous earth, cellulose filter, followed by 3 g of each biomass and another layer of cellulose filter. The extraction was carried out using tap water (pH 6.35 ± 0.33), constant pressure (1500 psi) and solid:liquid ratio of 1:11, considering the influx of new solvent per extraction cycle. Isothermal s-SWE was carried out at two conditions of fixed temperatures (120 and 150 °C) and consecutively increasing extraction times (10. 20 and 30 min) were selected as extraction variables. The resulting fractions from s-SWE (LF-10′, LF-20′, and LF-30′), as shown in the schematic extraction representation (Fig. 1a), for each byproduct and were dried in freeze-dryer (FreeZone 6, Labcombo, USA) before characterization. Extractions were performed in duplicates for each condition and vegetable side-stream.

### 3.2 Low-pressure hot water extraction (LPHW)

A comparative LPHW method was performed using an autoclave CertoClav Classic (CertoClav Sterilizer GmbH, AT) at 125 °C and 140 °C. Biomass powders were kept in a solid liquid ratio of 1:20 to avoid solvent saturation during the static extraction procedure, where no new influx of solvent is added to the extraction batch. The extraction was carried out at fixed time (15 and 30 min for each temperature), using tap water (pH 6.35 ± 0.33) as solvent. Pressure ranged according to experimental temperature, which 125 °C corresponds to 20.3 Psi and 140 °C to 39.1 Psi. Extracts (LF-15′ and LF-30′), as shown in the schematic extraction representation (Fig. 1a), for each byproduct were recovered by centrifugation (8000 rpm at 4 °C for 15 min) and dried in freeze-dryer (FreeZone6, Labconco Co., Missouri, USA) before characterization. Extractions were performed in duplicates for each condition and vegetable side-stream.

### 3.3 Physicochemical characterizations of biomasses and extracts

**Moisture content:** was determined gravimetrically and the ratio with its initial mass was calculated after 48 h drying in a FreeZone6 freeze-dryer (Labconco Co., Missouri, USA).

**Ash content:** was determined by the calcination of biomass following the standard method of AOAC (1990).

**Total lignin content:** Lignin content in the raw biomasses was determined following the International Organization for Standardization method 21,436 (International Organization for Standardization, 2022). Briefly, the biomasses were submitted to a two-step hydrolysis with sulfuric acid. The insoluble fraction was quantified as Klason lignin, while the soluble lignin was measured in the hydrolysate by spectrophotometry (Genesys 10uv, Thermo Fischer, USA) at 205 nm. The concentration of soluble lignin was calculated using an absorptivity factor of 110 L·g^−1^·cm^−1^ at 205 nm. Total lignin content was determined as the sum of Klason and soluble lignin. All analyses were performed in triplicate for each biomass.

**Global yield**: Global extraction yield (Y) was gravimetrically determined in function of the dry extract mass (We) in relation to the initial dry biomass weight (Wb).

**Total starch content:** Starch content in the prepared side stream extracts was evaluated using Megazyme total starch kit (Wicklow, IE). The measurements were carried out in a microplate reader FLUOstar Omega (BMG Labtech, GE) at 510 nm and using glucose as standard.

**Carbohydrate content:** The total carbohydrates content was evaluated in terms of the monosaccharide composition obtained by acid hydrolysis. For biomasses, sulfuric acid hydrolysis following the method described by Saeman et al. (1954) for determination of total neutral sugars content. Besides, a two-steps methanolysis followed by trifluoracetic acid (TFA) hydrolysis was applied to investigate hemicellulose and uronic acid content in the biomasses and in the different PLE extracts, in accordance with the method of Willför et al. (2009). Determinations were performed in a high-performance anion-exchange chromatography coupled to pulsed amperometric detection (HPAEC-PAD) using a Dionex ICS-6000 system (Thermo Fischer, USA). A column CarboPac™ PA20 (3 × 150 mm, Thermo Fischer, USA) was used for separation at 0.4 mL.min^−1^. The gradient method consisting of Milli-Q water, 200 mM NaOH, and 100 mM NaOH +100 mM NaOAc, as eluents A, B, and C, respectively as following: equilibration for 8 min with 1.2 % of B; 20 min of 1.2 % B; 10.1 min of 50 % B; 15.9 min of 100 % C; 3.9 min of 100 % B; 10.1 min of 1.2 % B. Quantification was carried out against calibration curve built with neutral sugars (fucose, arabinose, rhamnose, galactose, glucose, xylose, and mannose) along with uronic acids (galacturonic and glucuronic acids).

**Crude and soluble proteins:** The crude protein content present in the biomasses was evaluated in terms of total nitrogen content using an elemental analyzer (Rapid N Exceed, Elementar, Langenselbold, DE). The results were corrected by the 6.25 factor to obtain crude protein values. Quantification of soluble proteins in the extract was assessed by the method described by Bradford (1976) using a Bio-Rad (Hercules, USA) protein assay kit. A standard curve built using albumin (bovine origin, fraction V, 98 % PA; Sigma-Aldrich, SE) with the measurements performed at 595 nm.

**Total phenolic compounds (TPC):** TPC of biomasses was evaluated as the sum of free and bonded phenolics. Free phenolics were first extracted using an aqueous-organic method as described by Pérez-Jiménez et al. (2008). The residual biomasses were then subjected to saponification with 2 M sodium hydroxide at 60 °C overnight, followed by ethyl acetate partition at a 1:2 ratio (sample:ethyl acetate), repeated three times, to obtain the bonded phenolic fractions. For the extracts, TPC was determined by resuspending 10 mg/mL of each extract in ddH₂O. The evaluation of TPC for both raw biomasses and obtained extracts was performed based on their interaction with Folin–Ciocalteu's reagent, using an adaptation of the method described by Cicco et al. (2009). Briefly, 25 μL of extracts or biomasses and 25 μL of Folin-Ciocalteu reagent were added to 96-well microplate. After 2 min, 200 μL of sodium carbonate (5 %; w:v) was added. Microplates were incubated at 37 °C in the dark for 30 min and read at 760 nm in a FLUOstar Omega (BMG Labtech, GE) microplate reader. Gallic acid was used as standard, and the results were expressed in mg GAE.g^−1^ of dry sample.

**Phenolic compounds screening by HPLC-DAD:** The profiling of phenolic compounds in the biomasses and extracts was carried out by high-performance liquid chromatography (HPLC) using a Waters HPLC-DAD system constituted by a separation module (Waters 2695, USA) coupled to a photodiode array detector (Waters 1996, USA). A ZORBAX StableBond C18 column (Agilent Technologies, USA) was used for separation. A gradient method was performed with 1 % acetic acid (*v*/v) and acetonitrile as eluents A and B, respectively. The elution gradient was used as follow: 0–12 min (95 % A) 12–23 min (90 % A), 23–25 min (50 % A), 26–28 min (35 % A), 28–45 min (95 % A) in a solvent flow of 1.0 mL/min and injection volume of 25 μL. Calibration curve was performed using hydroxybenzoic acids (gallic, gentisic, and valinic acids), hydroxycinnamic acids (caffeic, ferulic, sinapic, p-coumaric and cinnamic acids) and flavonoids (catechin, rutin, myricetin, luteolin, quercetin, and kaempferol) at concentrations of 0.005–0.1 g-L^−1^ and used for quantification of compounds. Individual phenolic acids and flavonoids were relatively quantified based on the retention times of the standards at 325 nm, apart from catechin and kaempferol which was determined at 375 nm. Prior to analysis, biomasses were saponified with 2 M sodium hydroxide at 60 °C overnight, whereas extracts were saponified at 30 °C overnight, as described for the determination of TPC. Samples were then resuspended in a 50:50 mixture of eluents A and B and injected into the system. For biomasses, only the phenolic acids profile was determined.

### 3.4 In vitro antioxidant activity

*Scavenging activity against radical DPPH:* Evaluation of scavenging activity against radical DPPH (1,1-diphenyl-2-picrylhydrazyl) was carried out according to the method proposed by Brand-Williams, Cuvelier & Berset (1995) with few adaptations to the micro assay. In brief, 100 μL of samples at 10 mg/mL resuspended in ddH_2_O, or standards are mixed with 100 μL of the radical DPPH 0.2 mM and reacted in the dark for 30 min. Microplates were read in FLUOstar Omega (BMG Labtech, GE) with the absorbance at 517 nm at the time 0 and 30 min later. Additionally, the assay was applied to the raw biomasses to evaluate their antioxidant performance. For this, a dispersion of raw biomass (10 mg/mL) was prepared in methanol, homogenized for 1 h at 600 rpm at room temperature, and then centrifuged, with the supernatant used for analysis.

*Scavenging activity against radical ABTS*: ABTS (2′2-azinobis (3-ethyl-benzothiazoline-6-sulphonate) radical scavenging activity of extracts was estimated according to the method described by Re et al. (1999) with minor modifications. Briefly, samples and standards concentrations were adjusted to have less than 80 % of the absorbance of the control. The reaction was carried out by adding to 20 μL of the sample, 200 μL of radical ABTS 7 mM, previously prepared in 2.45 mM of potassium persulfate and diluted with ethanol. Microplates were incubated in the dark at 30 °C for 25 min, and read in the microplate reader FLUOstar Omega (BMG Labtech, GE) with absorbance at 734 nm.

### 3.5 Antimicrobial activity

The antimicrobial activity of extracts against representative strains of typical food pathogens including a Gram-negative *Escherichia coli* (CCUG 10979) and two Gram-positive *Listeria innocua* (CCUG 15531 T) and *Bacillus cereus* (CCUG 7414) were investigated. Vegetable extracts were resuspended in DMSO 10 % (v:v) in the range of 100–6.25 mg/mL. *E. coli* and *L. innocua* strains were cultivated in tryptic soy broth (TSB), while *B. cereus* was cultivated in Lysogenic broth (LB) at 37 °C for 24 h. Optical density at 600 nm (OD_600_) of microorganism media was adjusted to McFarland 0.5 standard and diluted to approximately 10^5^ colony forming units [CFU]/mL. Bacterial media was used to inoculate different concentrations of extracts followed by incubation for 24 h at 37 °C. The minimum inhibitory concentration (MIC), which is the lowest concentration of extract that can inhibit bacterial growth, was qualitatively determined.

### 3.6 Statistical analysis

Statistical significance was calculated using one-way Analysis of Variance (ANOVA) with a significant level of 5 %. When ANOVA results were significant, a Fisher's LSD post-hoc test was used to compare group means. The statistical analysis was carried out using R Statistical Software (v4.5.0; R Core Team 2025). The relationships among different PLE types, extraction conditions, and biomasses over the bioactivity of the extracts obtained were assessed by multivariate analysis, specifically Principal Component Analysis (PCA). PCA was carried out on software OriginPro 2023 (Originlab Corporation, MA, USA).

## 4 Results & discussion

### 4.1 Raw vegetables side streams characterization

Understanding the composition of the raw material is fundamental for fine-tuning the extraction of target compounds. The basic chemical composition of different biomasses is described in Table 1. All biomasses exhibited high moisture content, ranging from 85.11 % in artichoke leaves to 91.80 % in cauliflower leaves, resulting in relatively low dry matter values. Spinach leaves had the highest mineral content (*p* < 0.05), as reflected in their ash content (200.27 ± 1.27 mg·g^−1^ DW), a value consistent with that reported by El-Sayed (2020) for spinach powder. Ash content represents the inorganic fraction, including minerals essential for human consumption. Similar values of ash content have also been reported for artichoke (Salem et al., 2015), broccoli (Campas-Baypoli et al., 2009), and cauliflower (Mythili et al., 2021) by-products.

**Table 1:** Chemical composition of distinct raw vegetables matrices.

Compounds	Artichoke	Broccoli	Cauliflower	Spinach
Moisture content (mg.g^−1^ FW)	811.10 ± 0.90^c^	879.40 ± 0.20^b^	917.70 ± 0.41^a^	884.50 ± 0.97^b^
Ash (mg.g^−1^ DW)	149.67 ± 0.81^b^	105.39 ± 5.38^c^	106.34 ± 0.78^c^	200.27 ± 1.27^a^
Carbohydrate content (mg.g^−1^ DW)	566.33 ± 72.96^a^	508.95 ± 47.81^ab^	585.51 ± 30.39^a^	441.90 ± 58.78^b^
Starch (mg.g^−1^ DW)	121.94 ± 6.97^a^	47.53 ± 10.02^c^	65.06 ± 4.74^b^	18.61 ± 2.52^d^
Total lignin (mg.g^−1^ DW)	131.28 ± 13.95^a^	109.51 ± 4.56^b^	62.21 ± 2.70^c^	125.42 ± 0.19^a^
Crude protein (mg.g^−1^ DW)	148.32 ± 13.72^c^	254.49 ± 25.40^a^	160.54 ± 6.98^bc^	179.95 ± 10.71^b^
Soluble protein (mg.g^−1^ DW)	19.4 ± 2.17^b^	16.26 ± 0.70^bc^	13.36 ± 0.23^c^	85.25 ± 4.14^a^
Total phenolic compounds (mg.g^−1^ DW)^1^	14.74 ± 0.44^b^	13.47 ± 0.55^c^	10.51 ± 0.61^d^	17.75 ± 0.78^a^
Free phenolic acids (mg.g^−1^ DW)	7.26 ± 0.45^b^	4.82 ± 0.70^c^	4.55 ± 0.71^c^	10.61 ± 0.04^a^
Phenolic acids (mg.g^−1^ DW)	1.87 ± 0.18^a^	0.46 ± 0.02^c^	0.86 ± 0.21^b^	0.29 ± 0.03^c^

DW refers to dry weight and FBW refers to fresh weight.

Results are expressed in mean standard deviation. Letters in the same line represent statistically different (*p* < 0.05) by Fisher's test.

1 Determined as the sum of free and bonded phenolics by Folin-Ciocalteu assay.

The different vegetable side streams are a rich source of carbohydrates and proteins. Artichoke leaf shows a high content of carbohydrates, which among the biomasses has the highest content (p < 0.05) of starch (121.94 ± 6.97 mg.g^−1^ DW). On the other hand, broccoli leaf presented the higher content (*p* < 0.05) of crude protein (254.49 ± 25.40 mg.g^−1^ DW), from which a low content of soluble protein is detected. In contrast, despite the moderate content of crude protein for spinach (179.95 ± 10.71 mg.g^−1^ DW), the biomass presented the high content of soluble proteins. Beyond macromolecules, the extraction of bioactive compounds with antioxidant or antimicrobial properties from by-products represents an attractive biorefinery strategy for applications in food, cosmetics, and materials. The total phenolic compounds (TPC) in the different biomasses ranged from 10.51 ± 0.61 to 17.75 ± 0.78 mg GAE.g^−1^, demonstrating their potential for valorization. Among phenolic compounds, phenolic acids are an important class present in these biomasses. The phenolic acid profile of the different biomasses is presented in Fig. S1. Artichoke leaves exhibited the highest phenolic acid content (p < 0.05), accounting for 12.68 % of the TPC and 25 % of the bound fraction of phenolic compounds. Ferulic acid and ρ-coumaric acid are the main hydroxycinnamic acids in all evaluated biomasses except broccoli leaves, which is mainly represented by sinapic and cinnamic acids.

### 4.2 PLE on recovery of bioactive extracts

In the present study the effect of PLE type was investigated on the recovery of functional extracts from distinct green leafy side streams. The effect of the processing type on the extracts was primarily evaluated in terms of extraction yield as shown in Fig. 1.

Overall, the extraction yield was influenced by the type of processing. For s-SWE, yields increased significantly (*p* < 0.05) with both temperature and the number of extraction cycles for all biomasses. In comparison, for LPHW, yields also tended to increase with higher temperature and longer extraction time, except for artichoke leaf extracts, which may indicate solvent saturation during single-step extraction. The yields obtained with LPHW at the highest temperature and time (140 °C, 30 min) were statistically similar to those achieved with s-SWE at the highest temperature and maximum number of cycles (150 °C, 30 min), except for artichoke leaves, for which the LF150–30′ extract from s-SWE yielded the highest value. Alternatively, when biomass fractionation is not the main objective, s-SWE may be a suitable extraction technique as it combines multiple extraction cycles to achieve higher yields than single-step LPHW, which may require some downstream processes for purification of the targeted compound. Additionally, one of the main drawbacks from SWE is the industrial-scale equipment, which is not totally well established yet making it difficult for upscaling solutions. Besides, in SWE the requirement for elevated temperatures and pressures increases the energy consumption of the technique (Todd & Baroutian, 2017).

**Fig. 2:**
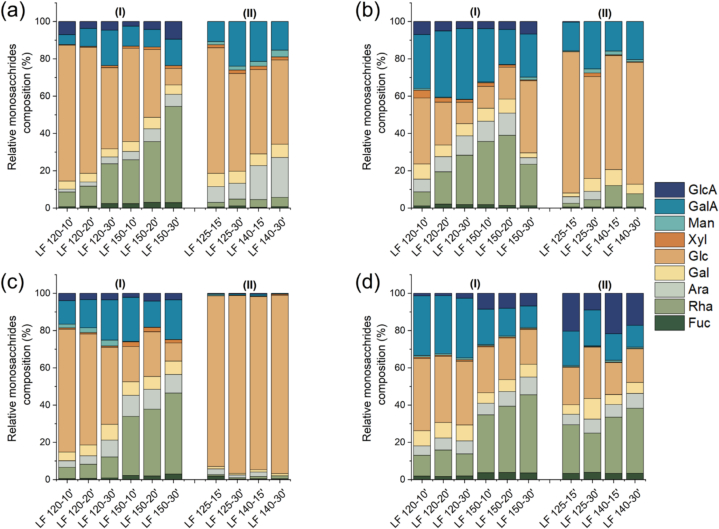
Relative monosaccharides composition from the different green extract leaves including artichoke (a), broccoli (b), cauliflower (c), and spinach (d), obtained by s-SWE (I) and LPHW (II) determined in terms relation to fucose, rhamnose, arabinose, galactose, glucose, xylose, mannose, galacturonic acid and glucuronic acid. (For interpretation of the references to colour in this figure legend, the reader is referred to the web version of this article.)

Green leaf extracts showed a high concentration of carbohydrates (Fig. S2), particularly in samples obtained by SWE compared with LPHW, except for artichoke extracts, where the extraction method had no statistically significant effect (*p* > 0.05) on carbohydrate content. This increase may be attributed to the hydrolytic power of SWE. An interesting result was observed in the carbohydrate profiles of the extracts (Fig. 2), in which a remarkable extraction of galacturonic acid, glucuronic acid, and rhamnose was observed for s-SWE compared to LPHW across all green leaves. This indicates that pectins are more readily extracted under higher-pressure conditions SWE. Under subcritical conditions, the ionic product of water increases with temperature and pressure, leading to higher concentrations of H^+^ and OH^−^ ions compared to ambient conditions (Kulkarni et al., 2022). Consequently, pectin extraction is facilitated through the cleavage of bonds anchoring pectins to the plant cell wall structure (Chandel et al., 2022), a process less likely to occur under the milder conditions of LPHW. Thus, s-SWE appears promising for pectin recovery as a green process, reducing the need for strong organic acids typically used for this purpose.

Furthermore, differences in pectin structure were observed between extracts obtained by s-SWE and LPHW (Fig. S2). An increase in the Rha/GalA ratio, associated with the main pectin chain, along with a decrease in the (Ara + Gal)/Rha ratio, associated with pectin side chains, suggests that SWE promotes cleavage of both the pectin backbone and the RG-I domain simultaneously (Zhi et al., 2017), potentially enhancing the extractability of pectic poly and/or oligosaccharides. Conversely, the higher (Ara + Gal)/Rha ratio observed with LPHW at lower extraction temperatures (125 °C) indicates the extraction of pectins with longer or more branched side chains for all green leaves except spinach, suggesting that milder extraction condition preserves these side branches. Overall, these findings indicate that both the extraction process and the type of biomass influence the length and branching of recovered pectins the side chains, which may, in turn, affect their techno-functionality (Assifaoui et al., 2024).

**Fig. 3:**
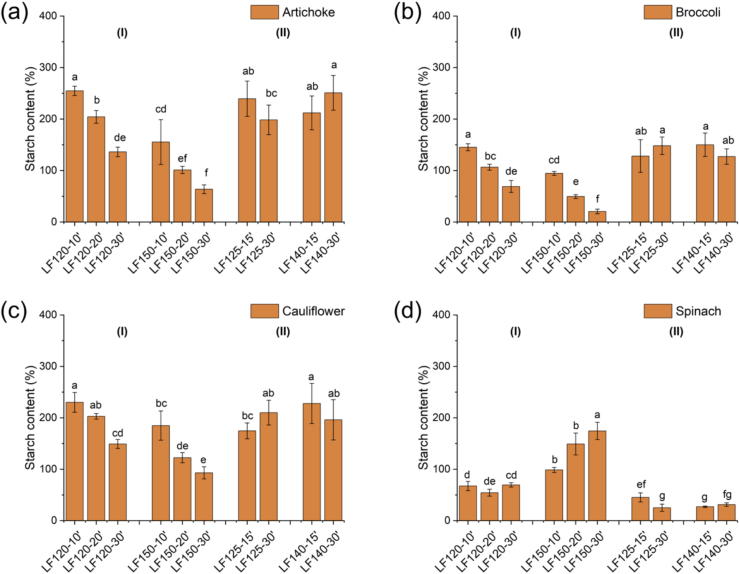
Starch content on the different green leaves extracts: artichoke (b), broccoli (c), cauliflower (c), and spinach (e) obtained by (I) s-SWE and (II) LPHW. Different letters above results indicate significant differences by Fisher's test. (For interpretation of the references to colour in this figure legend, the reader is referred to the web version of this article.)

Additionally, the starch content (Fig. 3), a non-structural carbohydrate, decreased significatively (*p* < 0.05) with the number of extraction cycles in s-SWE for all biomasses, except spinach leaf, where increased extraction severity (higher temperature and cycles) appears to favor the extraction of starch. This finding highlights that starch recovery as a target compound is biomass-dependent. In contrast, in single-step LPHW extractions, starch concentration remained nearly constant, with similarly high values (p < 0.05) compared with s-SWE performed at 120 °C for 10 and 20 min, for artichoke, broccoli, and cauliflower. Increasing extraction cycles in these biomasses, contributed to reducing the starch content due to the fractionation of this highly hydrophilic compound. A similar trend was previously reported by Rudjito et al. (2019) when applying s-SWE to wheat bran. These results demonstrate the potential of using sequential extraction to modeling and optimize the extraction processes, enabling recovery conditions to be tailored to target compound. Furthermore, residues obtained after extraction for both PLE processes were rich in cellulose as shown in Fig. S4, with a high glucose content of around 30 % in all the green leaf residues, along with potentially more recalcitrant hemicelluloses. These residues also hold potential for valorization approaches for cellulose recovery after the extraction of bioactive extracts (Ali et al., 2023).

Regarding the protein content in the green leaf extracts (Fig. S3), increasing the severity of s-SWE extraction favored protein recovery, particularly at 150 °C for two or three cycles, where significantly higher concentrations (*p* < 0.05) were obtained compared with mild s-SWE and LPHW conditions. Among the biomasses, spinach extracts exhibited higher protein content for both processing types. This effect was even more pronounced for SWE at the highest extraction temperature. A similar trend was reported by Nathia-Neves and Alonso (2024), who found that increasing the temperature from 125 °C to 150 °C improved protein recovery from sunflower by-products under SWE, indicating that higher temperatures enhance protein solubility. The high protein content in spinach extracts compared to other green leaf extracts aligns with the elevated levels of soluble proteins observed in the raw biomass (Table 1), where proteins are readily accessible. In this study, the protein content of the spinach extract obtained at s-SWE LF150–30′ was 193.84 mg·g^−1^, representing 46.85 % of the total proteins in the original biomass. This result is in accordance with the total amino acid content of 189.21 mg·g^−1^ reported by Nemzer et al. (2021) for raw spinach leaf biomass. These findings suggest that s-SWE effectively recovers a comparable amount of accessible proteins to that present in the raw biomass, highlighting its potential for protein biorefinery applications targeting alternative sources for the food sector.

Phenolic compounds are a class of bioactive compounds that can exhibit antioxidant and antimicrobial properties. In this study, the phenolic content of the obtained extracts ranged from 6.53 to 28.55 mg GAE.g^−1^ DW for LPHW and 5.34–27.86 mg GAE.g^−1^ DW for s-SWE across the different biomasses. Overall, 150 °C was the optimal temperature for green leafy vegetables side streams evaluated, as it enhanced the extraction efficiency of phenolic compounds without reducing the extraction yield in s-SWE. In contrast, LPHW at 125 °C for 30 min or 140 °C at independent of the time, provided high and comparable TPC values to those obtained by s-SWE at 150 °C for three extraction cycles. The maximum TPC recovered from each biomass was consistent with literature values for artichoke leaves extracted with methanol (Masci et al., 2025), broccoli leaves recovered using ethanolic ultrasound extraction (Cerro et al., 2025), and spinach leaves obtained from mild ultrasound extraction (Altemimi et al., 2015), while cauliflower leaf extracts showed lower values than those reported for methanol extracts (Zeb et al., 2022). Additionally, under the maximum extraction conditions for TPC, all extracts showed higher values compared with the raw biomasses (Table 1).

**Fig. 4:**
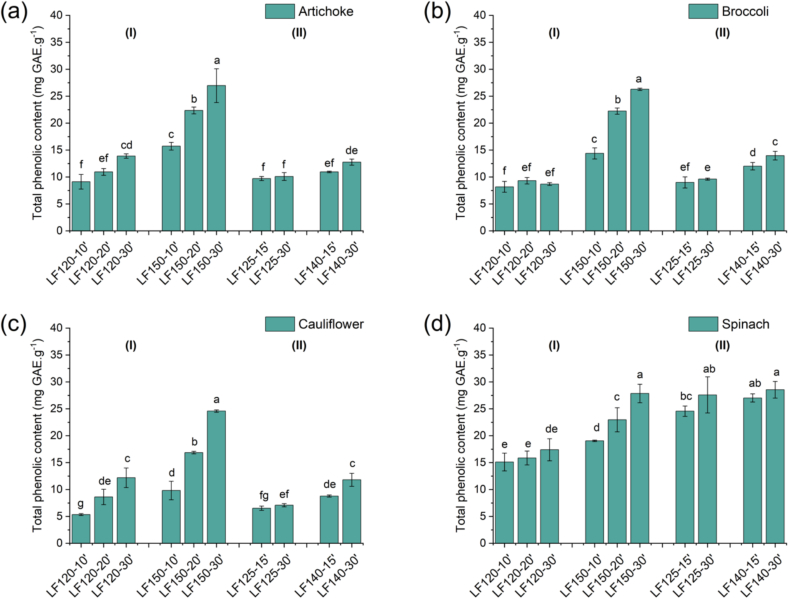
Total phenolic content from the different green leaf extracts from (a) artichoke, (b) broccoli, (c) cauliflower, and (d) spinach obtained by (I) s-SWE and (II) LPHW. Different letters above the results significant differences by Fisher's test.

For both the PLE types, increasing temperature and time generally favored the recovery of TPC, as shown in Fig. 4. This trend can be explained by the fact that phenolic compounds are readily extracted in their free form, along with the bonded phenolics attached to low-molecular-weight compounds, which do not require high energy transfer (Shahidi & Yeo, 2016). However, as process severity increases, the cleavage of ester and ether bonds linking phenolic compounds to structural components makes them more accessible, thereby enhancing their extraction (Cocero et al., 2018). This effect is more pronounced in s-SWE, where phenolic concentration increased not only with temperature but also the number of extraction cycles. This is an expected result, since in each cycle, the solvent is renewed, preventing the degradation of thermally labile phenolic compounds caused by prolongated heat exposure, while simultaneously mitigating mass transfer limitations that may arise from solvent saturation. Notably, spinach leaf extracts obtained by LPHW also showed a marked increase in TPC, indicating that both the choice of process and the type of biomass influence phenolic recovery.

**Fig. 5:**
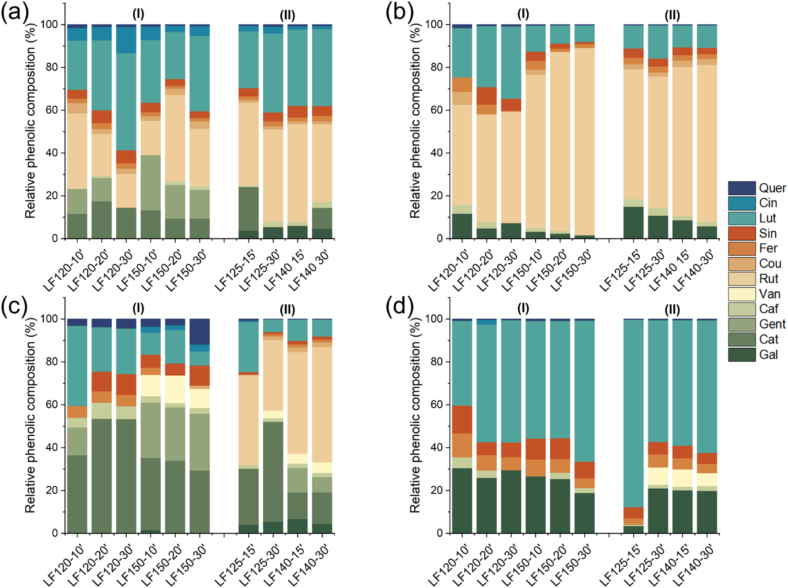
Relative phenolic composition from the different green extract leaves including (a) artichoke, (b) broccoli, (c) cauliflower, and (d) spinach, obtained by s-SWE (I) and LPHW (II) determined in relation to the presence of quercetin, cinnamic acid, luteolin, sinapic acid, ferulic acid, ρ-coumaric acid, rutin, vanillic acid, caffeic acid, gentisic acid, catechin, and gallic acid. (For interpretation of the references to colour in this figure legend, the reader is referred to the web version of this article.)

In addition to TPC quantification, phenolic compounds were identified by HPLC-DAD (Fig. 5). The extraction method influenced the phenolic composition among the different extracts, with extraction time and temperature also playing important roles. For artichoke leaf extracts, s-SWE was particularly effective in extracting catechin, gentisic acid, rutin, and luteolin, whereas LPHW facilitated the extraction of rutin more efficiently. In broccoli extracts, s-SWE promoted the recovery of rutin and luteolin at 120 °C, while a similar phenolic composition was obtained for s-SWE at high extraction temperatures and LPHW. In cauliflower leaf extracts, catechin and rutin were the main phenolics extracted at low temperature under LPHW, with rutin becoming dominant as temperature increased. In contrast, s-SWE primarily extracted catechin, followed by rutin at higher temperatures and longer extraction times. For spinach leaf extracts, the main change in phenolic profiles was the increase of gallic, ferulic, and sinapic acid content with s-SWE compared to LPHW.

These results show that by adjusting the properties of water as a solvent, through variation in pressure, temperature, extraction time, and the number of extraction cycles, it is possible to optimize the extraction process for specific target phenolic compounds. Overall, higher levels of phenolic compounds were observed in the extracts when measured by TPC compared with the amounts quantified through HPLC (Fig. S3). The discrepancy can be attributed not only to the non-specific nature of the Folin-Ciocalteu assay, as previously noted by Sánchez-Rangel et al. (2013), but also to the lack of standards for measuring phenolics within the highly complex structure of the vegetable side streams selected in this study, requiring a more advanced technique for identification and quantification.

### 4.3 Extracts bioactivity

The extracts were evaluated for their bioactivity in terms of free radical scavenging activity (Fig. S5), with results expressed as half maximal effective concentration (EC_50_ value). Recovering natural antioxidant extracts is an attractive approach for valorizing agri-food by-products, given the high content of these compounds in plant biomasses, where they are typically produced as secondary metabolites. Moreover, the growing concern over the use of synthetic antioxidants, particularly in food products, presents an opportunity for utilizing vegetables side streams as a source of natural additives to replace synthetic compounds. A close look at the antioxidant activity attributed to the biomasses (Fig. S1b) reveals that the extracts obtained by both PLE techniques presented higher antioxidant activity than their respective biomasses, since high values of EC_50_ are reported for the biomasses in relation to the extracts, showing that less extract amount is required to reduce by 50 % the free radical available. This result is expected, as bioactive compounds become more accessible upon extraction from the cell wall matrix, making them more available for free radical neutralization reactions, and therefore more active antioxidants.

Complementarily, the antimicrobial activity of the different extracts was assessed against common food-borne pathogens, and the results are shown in Table S1. Natural extracts are of interest due to their rich constitution in phytonutrients with a wide range of biological functions, including antimicrobial effects. These compounds represent an alternative to synthetic antimicrobials and might improve food quality by simultaneously reducing lipid oxidation and microbial growth (Nieto et al., 2023). Overall, all aqueous extracts required relatively high concentrations to inhibit bacterial growth, similar to findings for green leaves reported by Radünz et al. (2024), except for cauliflower extracts obtained by LPHW, which demonstrated remarkable antimicrobial activity. Relatively high values of MIC were also reported by Arslan (2024) using water as solvent for extraction of vegetable peels. Notably, artichoke leaf extracts demonstrated broad antimicrobial activity against both Gram-negative (*E. coli*) and Gram-positive (*B. cereus*) bacteria strains. Zhu et al. (2005) reported a minimum inhibitory concentration (MIC) of ethanolic extracts above 25 mg/mL against *E. coli* and 18 mg/mL against *B. cereus*. The main difference compared to the extracts obtained from artichoke using both PLE techniques may be due to the use of water as a solvent, which may not effectively extract certain compounds responsible for antimicrobial activity.

**Fig. 6:**
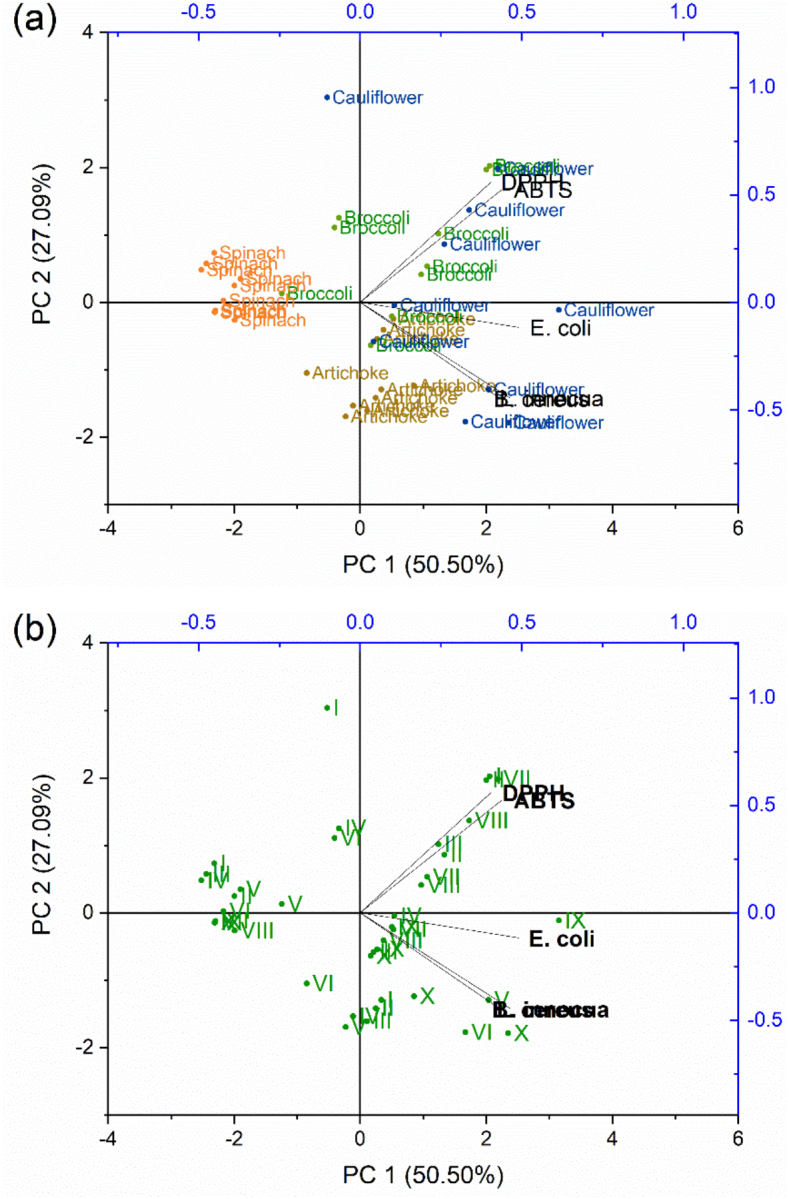
PCA biplot for the bioactivity of the different extracts obtained by PLE type in function of the biomass type (a) and in function of the extraction condition (b) where s-SWE is represented by I-III (LF120–10′, LF120–20′, LF120–30) and by IV-VI (LF150–10′, LF150–20′, LF140–30′), whereas LPHW is represented by VII-X (LF125–15′, LF125–30′, LF140–15′, and LF140–15′).

A Principal Component Analysis (PCA) was performed to better visualize the effect of PLE type and extractions conditions on the bioactivity of the different extracts. The first two principal components (PCs) accounted for 77.59 % of the total variance, with PC1 explaining 50.50 % and PC2 explaining 27.09 % of variance of the data as shown in Fig. 6. PC1 represented the main contrast, with the antioxidant activity (expressed as EC_50_ values for DPPH and ABTS radicals) loading negatively, and the antimicrobial performance in relation to *E. coli*, *B. cereus*, and L. *innocua* loading positively. Thus, samples with negative PC1 scores exhibited the lowest EC_50_ values, indicating high antioxidant activity, but low antimicrobial activities. Conversely, positive PC1 scores corresponded to antioxidant properties and stronger antimicrobial activity. PC2 reflected variation mainly in antimicrobial activity against Gram-positive bacteria (*B. cereus* and L. *innocua*), since *E. coli* contributed minimally to PC2 scores. Negative PC2 scores represented greater inhibition of Gram-positive bacteria, whereas positive scores indicated lower inhibition. On the other hand, the alignment of *E. coli* with PC1 seems to suggest that the effect of the extracts on Gram-negative strains may be related to the balance between antioxidant and antimicrobial activities.

Analyzing the effect of processing type and conditions on extract bioactivity, some distinction can be made. s-SWE samples (conditions I-VI) are clustered on the negative PC1 axis, indicating strong antioxidant properties but weak antimicrobial activity. Within s-SWE, progression from conditions I to VI tended to shift further along the negative PC1 axis, suggesting an improvement in antioxidant potential without marked changes in antibacterial activity. This trend might be explained by the increase in the concentration of phenolics compounds extracted per extraction cycle, as shown in Fig. 4, in comparison with the LPHW prepared extracts, suggesting the efficiency of s-SWE on the preparation of bioactive extracts. Besides, a predominance of LPHW samples (conditions VII-X) on positive PC1 side, reflects extracts with greater antimicrobial activity but reduced antioxidant activity.

The biomass type also influenced the distribution of the extracts, highlighting its role in modulating the process, as observed for protein, starch and TPC results. Spinach extracts tended to cluster in the negative PC1 region, indicating a pronounced antioxidant character but poor antibacterial activity, regardless of extraction condition or processing type. This may be attributed to the higher carbohydrate and protein/peptide content in the extracts, which could serve as carbon and nitrogen sources for microbial growth rather than inhibiting it. In contrast, methanolic extracts of spinach leaves obtained by ultrasound-assisted extraction have shown higher antimicrobial activity against Gram-negative bacteria (Altemimi et al., 2017). As noted by Deshmukh and Gaikwad (2024), the antimicrobial activity of natural extracts is highly dependent on their structural composition, which may explain the lower antimicrobial activity observed here when using water as the extraction solvent, regardless of the technique applied.

Cauliflower extracts, particularly those obtained by LPHW, tended to cluster toward the positive PC1 quadrant, which is associated with higher antibacterial activity. This trend is followed by broccoli leaf extract also obtained by LPHW. Artichoke extracts showed intermediate behavior within the s-SWE group, with both antimicrobial and antioxidant activities increasing alongside temperature and extraction cycles. The differences in antimicrobial activity among extracts likely reflect changes in phenolic profiles between extraction methods, as observed by HPLC. Additionally, variations in extract composition, driven by the solvent's ability to recover compounds of different polarity, appear to influence antimicrobial effectiveness. Overall, the PCA results suggest that both PLE methods (LPHW and s-SWE), together with processing conditions and biomass type, play a key role in tuning extraction parameters, underscoring the importance of aligning these factors with the desired bioactivity profile.

## 5 Conclusion

Green leaf extracts were obtained through pressurized liquid extraction using water as solvent, and the resulting fractions exhibited promising antioxidant and antimicrobial activities, highlighting their potential for use as both direct and indirect food preservation methods. The processing method influenced extract composition. Particularly, s-SWE produced fractions with distinct profiles, influenced by elevated temperatures and the use of multiple extraction cycles, likely due to enhanced access to intracellular compounds that could remain partially inaccessible during single-step extractions. Higher extraction temperatures favored phenolic compounds release, as evidenced by TPC and HPLC-DAD analyses, and this was accompanied by an increase in the antioxidant activity of the extracts. Additionally, s-SWE promoted the extraction of pectins with shorter or less branched side chains, whereas the milder LPHW process, despite yielding smaller amounts of pectins, preserved the side-chain integrity. No significant effect of the processing method on protein extractability was observed. However, notable differences were found among the biomass types, with spinach leaf standing out as the most promising source for protein recovery. Antimicrobial activity was also influenced by the processing method, with LPHW extracts demonstrating stronger antimicrobial effects. In contrast, s-SWE primarily enhances antioxidant properties; however, depending on the biomass type, such as artichoke, the resulting extracts can exhibit an intermediate profile, balancing antioxidant activity with antimicrobial performance. Overall, these findings highlight green leafy vegetables as valuable raw materials for the sustainable extraction of bioactive compounds with potential applications in food systems.

## CRediT authorship contribution statement

**Pamela F.M. Pereira:** Writing – review & editing, Writing – original draft, Visualization, Methodology, Investigation, Funding acquisition, Formal analysis, Data curation, Conceptualization. **Amparo Jiménez-Quero:** Writing – review & editing, Writing – original draft, Validation, Project administration, Methodology, Investigation, Funding acquisition, Conceptualization.

## Declaration of generative AI and AI-assisted technologies in the writing process

During the preparation of this work the authors used Chat-GPT 4.0 and Grammarly in order to improve linguistic quality and readability of the manuscript. After using this tool/service, the authors reviewed and edited the content as needed and take full responsibility for the content of the publication.

## Declaration of competing interest

The authors declare the following financial interests/personal relationships which may be considered as potential competing interests: Amparo Jimenez Quero reports financial support was provided by Chalmers University of Technology. Pamela Freire de Moura Pereira reports financial support was provided by Chalmers University of Technology. If there are other authors, they declare that they have no known competing financial interests or personal relationships that could have appeared to influence the work reported in this paper.

## Data Availability

Data will be made available on request.

## Appendix A Supplementary data

Supplementary material: Supplementary data contains analysis of total carbohydrates, phenolic compounds, soluble proteins and antioxidant activity. Image 1
