# Supplementary material for: Circular valorization of green leafy side streams via pressurized liquid extraction techniques for recovery of bioactive compounds
Source: Food Chem X. 2025 Aug 23;30:102944. doi: 10.1016/j.fochx.2025.102944 (PMC12410531; doi:10.1016/j.fochx.2025.102944)
Supplement: Supplementary file 1 — Supplementary material: Supplementary data contains analysis of total carbohydrates, phenolic compounds, soluble proteins and antioxidant activity. [file mmc1.docx]

**Supplementary Information**

**Circular valorization of green leafy side streams via pressurized liquid extraction techniques for recovery of bioactive compounds**

Pamela F. M. Pereira, Amparo Jiménez-Quero

Division of Industrial Biotechnology, Department of Life Sciences, Chalmers University of Technology, 412 96, Gothenburg, Sweden.

|  |  |
| --- | --- |

**Figure S1**. (a) Some phenolic acid compounds p including cinnamic acid (Cin), sinapic acid (Sin), ferulic acid (Fer), ρ-coumaric acid (Cou), caffeic acid (Caf) and gallic acid (Gal), and (b) radical scavenging ability of distinct green leaves raw materials

| 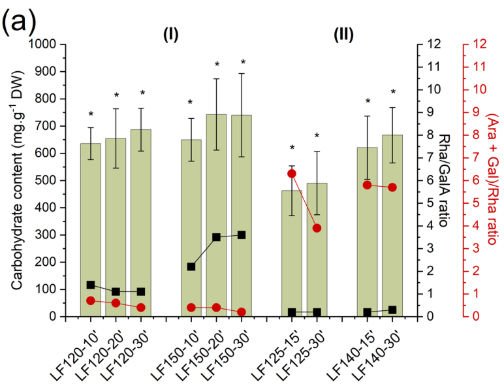 | 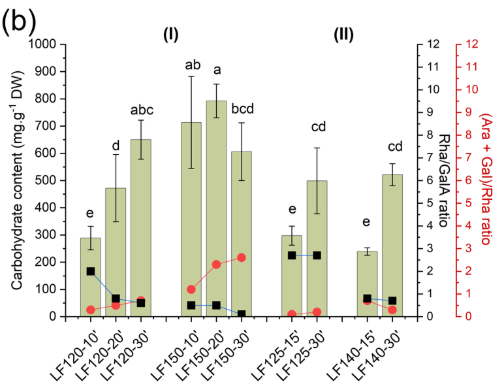 |
| --- | --- |
| 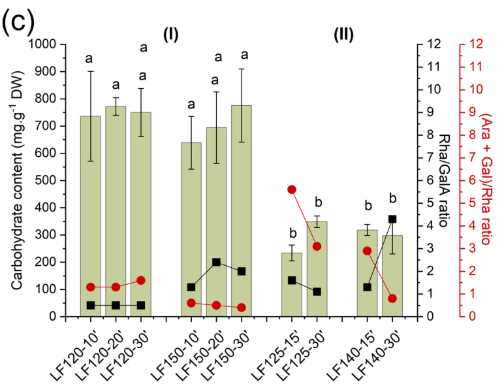 | 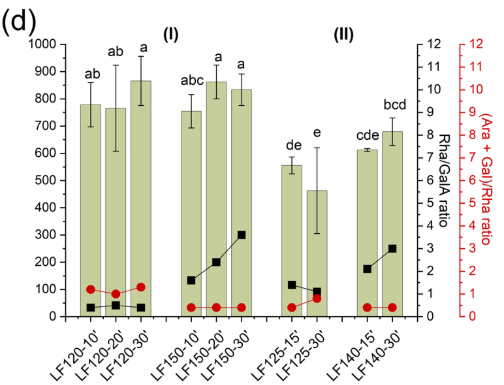 |

**Figure S2.** Total carbohydrate content and pectin substitution ratios Rha/GalA and (Ara+Gal)/Rha for the different vegetable side streams extracts obtained by (a) LPHW and (b) s-SWE.

| 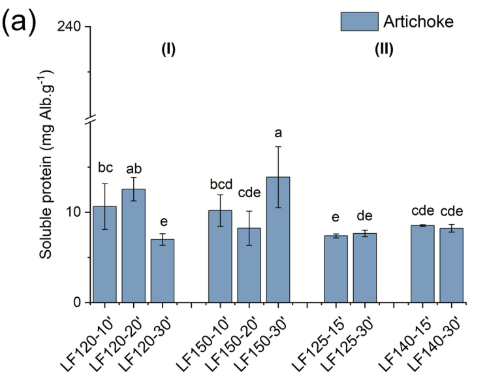 | 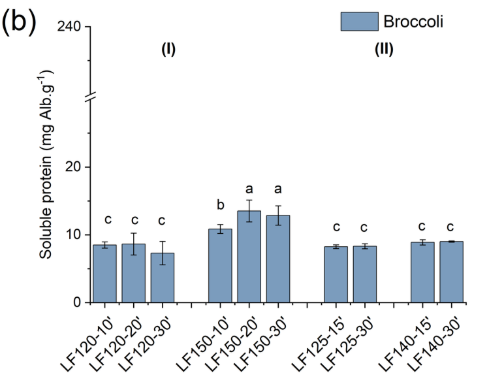 |
| --- | --- |
| 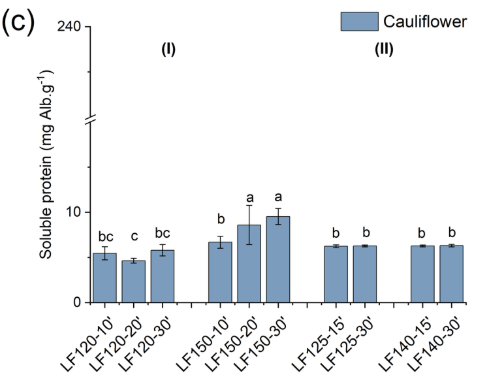 | 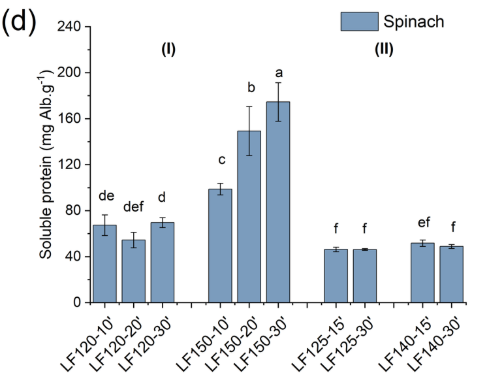 |

**Figure S3.** Soluble protein content from the different green leaf extracts from (a) artichoke, (b) broccoli, (c) cauliflower, and (d) spinach obtained by (I) s-SWE and (II) LPHW

| 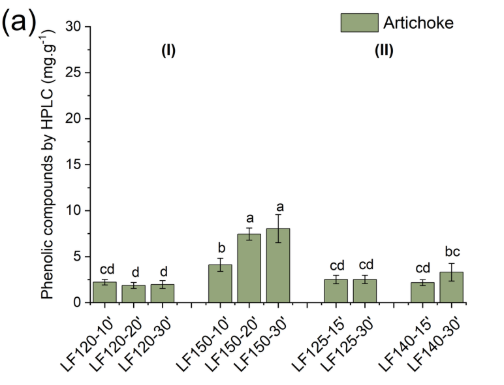 | 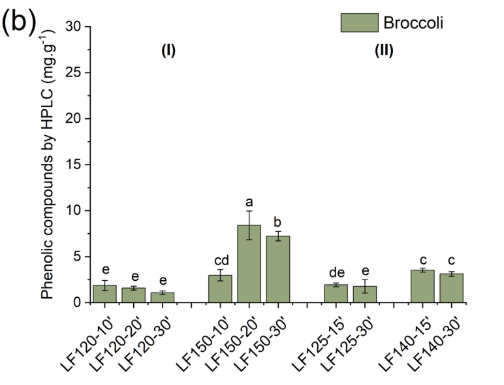 |
| --- | --- |
| 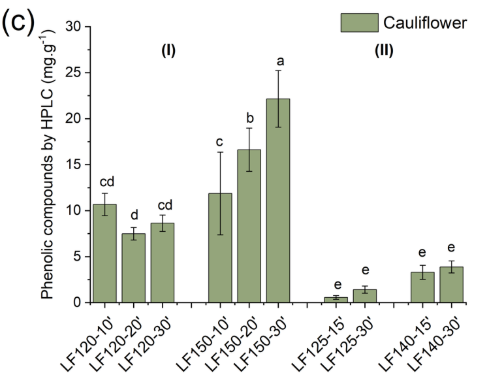 | 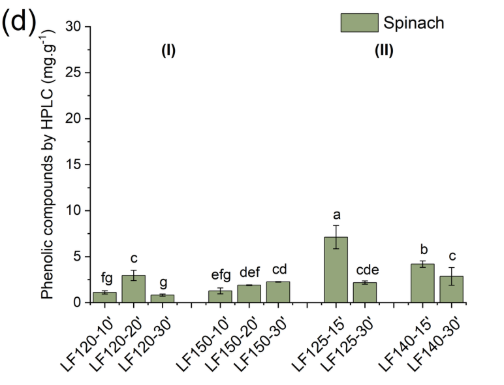 |

**Figure S3.** Phenolic compounds determined by HPLC-DAD from the different green leaf extracts from (a) artichoke, (b) broccoli, (c) cauliflower, and (d) spinach obtained by (I) s-SWE and (II) LPHW

| 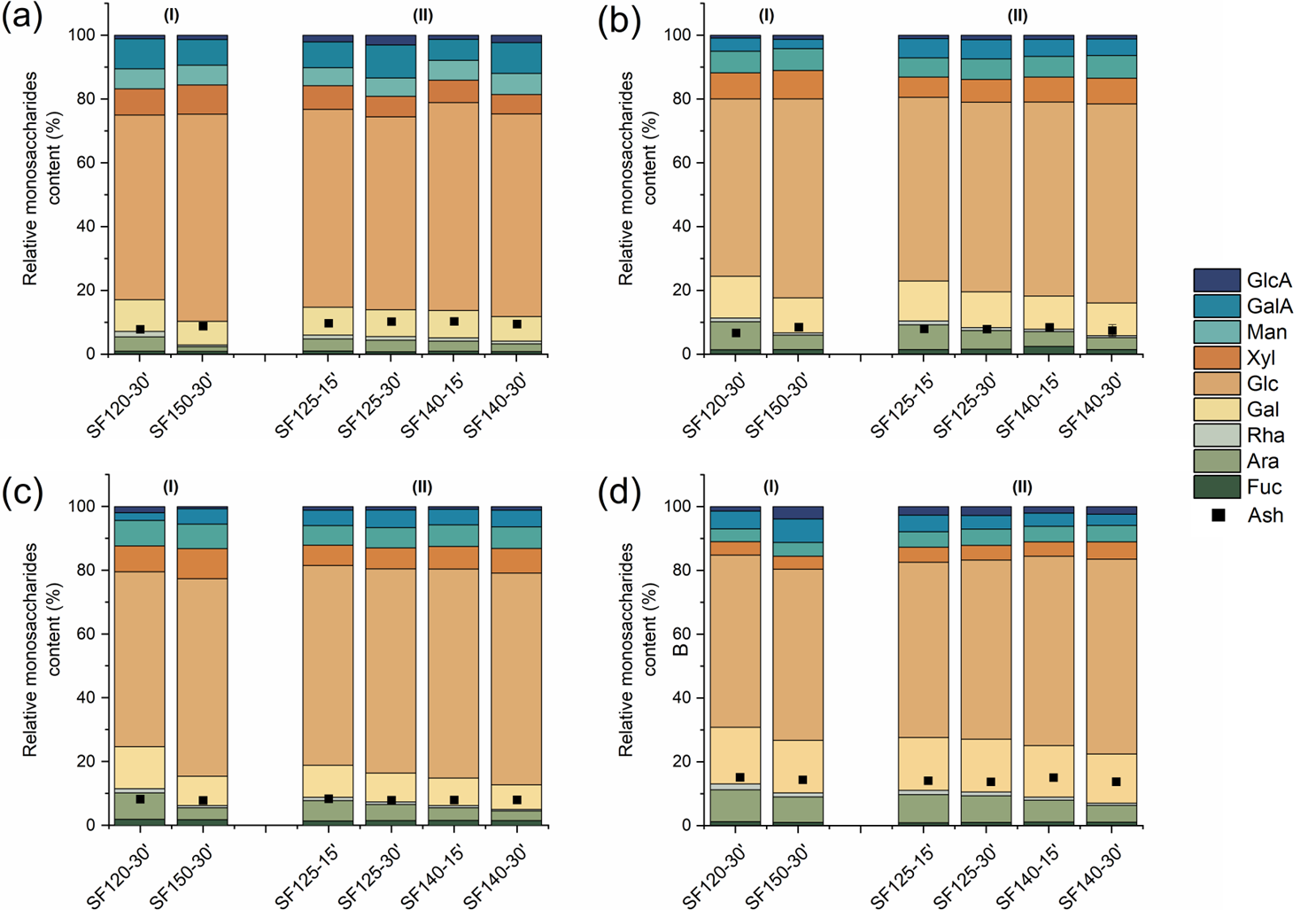 |
| --- |

**Figure S4.** Carbohydrate profile as the sum of neutral sugars determined by sulfuric acid hydrolysis and uronic acids determined by TFA hydrolysis, as well as the ash content from the different solid fractions (SF) obtained after extraction of the different green leaves artichoke (a) broccoli (b), cauliflower (c) and spinach (d) applying s-SWE (I) and LPHW (II) as processing extraction. Carbohydrate content is expressed in terms of fucose (Fuc), arabinose (Ara), rhamnose (Rha), galactose (Gal), glucose (Glc), xylose (Xyl), mannose (Man), galacturonic acid (GalA), and glucuronic acid (GlcA)

|  |  |
| --- | --- |
|  |  |

**Figure S5.** Radical scavenging behavior of different vegetable side streams extracts including (a) artichoke, (b) broccoli, (c) cauliflower, and (d) spinach obtained by s-SWE (I) and LPHW (II). Different letters above extracts from the same biomass and free radical indicate significant differences by Fisher’s test.

| **Table S1.** Minimum inhibitory concentration for vegetables extracts obtained under low pressure hot water extraction (LPHW) and sequential subcritical water (s-SWE) extraction conditions against different bacteria strains | | | | | |  |
| --- | --- | --- | --- | --- | --- | --- |
|  |  |  |  |  |  |  |
| **Extraction temperature (ºC)** | **Time (min)** | **Artichoke** | **Broccoli** | **Cauliflower** | **Spinach** |  |
| **LPHW** | | | | | |  |
|  | *E. coli* | | | | |  |
| LF125’ | 15 | 50 | 50 | 50 | >100 |  |
|  | 30 | 50 | 50 | 50 | >100 |  |
| LF140’ | 15 | 50 | 50 | 50 | >100 |  |
|  | 30 | 50 | 50 | 25 | >100 |  |
|  | *B. cereus* | | | | |  |
| LF125’ | 15 | 50 | 50 | 50 | >100 |  |
|  | 30 | 50 | 50 | 50 | 100 |  |
| LF140’ | 15 | 50 | 50 | 12.5 | >100 |  |
|  | 30 | 50 | 50 | 12.5 | >100 |  |
|  | *L. innocua* | | | | |  |
| LF125’ | 15 | 100 | 100 | 100 | >100 |  |
|  | 30 | 100 | 100 | 100 | 100 |  |
| LF140’ | 15 | 100 | 100 | 50 | >100 |  |
|  | 30 | 50 | 100 | 50 | >100 |  |
| **s-SWE** | | | | | |  |
|  | *E. coli* | | | | |  |
| LF120’ | 10 | 50 | 25 | >100 | >100 |  |
|  | 20 | 50 | 25 | 50 | >100 |  |
|  | 30 | 50 | 25 | 50 | >100 |  |
| LF150’ | 10 | 50 | 25 | 100 | >100 |  |
|  | 20 | 50 | 25 | 25 | >100 |  |
|  | 30 | 50 | 25 | 25 | >100 |  |
|  | *B. cereus* | | | | |  |
| LF120’ | 10 | 25 | 100 | 50 | 100 |  |
|  | 20 | 25 | 100 | 50 | 100 |  |
|  | 30 | 25 | 100 | 50 | 100 |  |
| LF150’ | 10 | 100 | >100 | 100 | >100 |  |
|  | 20 | 100 | >100 | >100 | 100 |  |
|  | 30 | 100 | >100 | >100 | 100 |  |
|  | *L. innocua* | | | | |  |
| LF120’ | 10 | 100 | 100 | >100 | >100 |  |
|  | 20 | 100 | 100 | 100 | >100 |  |
|  | 30 | 100 | 100 | 100 | >100 |  |
| LF150’ | 10 | 50 | >100 | 50 | >100 |  |
|  | 20 | 50 | >100 | 50 | >100 |  |
|  | 30 | 100 | >100 | 50 | >100 |  |
| Concentration in mg/mL | |  |  |  |  |  |
